# The Courage to Care: Teacher Compassion Predicts More Positive Attitudes Toward Trauma-Informed Practice

**DOI:** 10.1007/s40653-022-00486-x

**Published:** 2022-09-17

**Authors:** Catriona O’Toole, Mira Dobutowitsch

**Affiliations:** 1 Department of Education, Maynooth University, Maynooth, Co Kildare Ireland; 2 Marino Institute of Education, Griffith Avenue, Dublin 9, Ireland

**Keywords:** Teacher wellbeing, Stress and burnout, Ireland, Compassion, Trauma-informed practice

## Abstract

**Purpose:**

With greater awareness of the prevalence and impact of childhood trauma and adversity, teachers are now assuming a more active role in creating emotionally healthy environments and responding to student distress. However, supporting trauma-affected students can be a source of amplified teacher stress. Compassion has been identified as a promising construct for frontline professionals in terms of promoting psychological wellbeing, and increasing the sensitivity to detect, tolerate and respond to distress in others. It has also been identified as an important aspect of trauma-informed practice. Nevertheless, the role of compassion in teachers’ attitudes towards, and readiness for implementing trauma-informed practices has not yet been explored. This study aimed to address this gap.

**Methods:**

A sample of 377 primary and post-primary teachers in Ireland completed the Attitudes Related to Trauma-Informed Care Scale, the Professional Quality of Life Scale, the Self-Compassion Scale, and a socio-demographic survey.

**Results:**

Teachers were found to hold generally positive attitudes toward trauma-informed care. They exhibited low to moderate levels of secondary traumatic stress and burnout, and notably high levels of compassion satisfaction, indicating that they tend to embrace their caring role and find meaning and purpose in their work. Regression analyses showed that compassion satisfaction was the strongest predictor of positive attitudes toward trauma-informed care, followed by self-compassion. Older teachers were more likely to display positive attitudes toward trauma-informed care, whilst teachers in single-sex boys’ schools held attitudes that were less favorable.

**Conclusion:**

This study suggests potential benefits for both teachers and students of positioning compassion at the center of educational policy and practice. The results are contextualized within the Irish and international educational landscape.

## Introduction

Childhood trauma is common and can have devastating and wide-ranging impact on children’s health, wellbeing, and educational progression (Bebbington et al., 2009; Felitti et al., 1998; Dube et al., 2001). It is estimated that up to two-thirds of children have experienced at least one traumatic event (Felitti et al., 1998; Finkelhor et al., 2015), and these estimates only include exposure to events that are ostensibly traumatic (e.g., abuse, violence); many more children face continuous and repeated very negative experiences embedded in the relationships, discourses or structures of their lives, such as poverty, discrimination and oppression (Johnstone et al., 2018; O’Toole, 2022a).

Children exposed to trauma or adversity often have disrupted capacity for emotional regulation and difficulty navigating interpersonal relationships, which may manifest as disorganized behavior in the classroom (Brunzell et al., 2016; Crozier & Barth, 2005; Treisman, 2017). Their behavioral outbursts and/or emotional withdrawals can pose challenges for teachers who may not be aware of how trauma and adversity affect mind, body and behavior, and who have competing classroom demands (O’Toole, 2022a). Teachers can misinterpret children’s trauma responses as willful defiance, a lack of respect, or disengagement (Chafouleas et al., 2016; Thomas et al., 2019), which can result in punitive or confrontational responses. Unsurprisingly then, it can be difficult for these children to feel a sense of safety and connectedness at school, which in turn contributes to lower academic attainment and higher rates of absenteeism over time (Bellis et al., 2018).

### Trauma-Informed Practice in Education

Trauma-informed practice has been advocated in schools as a way to support teachers in understanding the nature and consequences of trauma and in building emotionally healthy classroom environments (Overstreet & Chafouleas, 2016; SAMHSA, 2014). It is a strengths-based approach that builds knowledge of the pervasive biological, psychological and social consequences of trauma with the ultimate aim of ameliorating, rather than exacerbating, their effects (Harris & Fallot, 2001; SAMHSA, 2014). Trauma-informed approaches provide a contextual understanding of the children’s behaviors, recognizing them as survival strategies rather than manifestations of disorder or deficit. While there are a variety of trauma-informed approaches, frameworks and strategies, the core underlying principles have been identified as safety, trustworthiness, collaboration, choice and empowerment, peer support, cultural humility and respect for diversity (Harris & Fallot, 2001; SAMHSA, 2014). Increasingly, trauma-informed approaches are recognized as important in mitigating the negative impact of trauma and promoting the growth and success of *all* students, but especially those affected by trauma (Brunzell et al., 2016; Dorado et al., 2016; Kim et al., 2021).

Whilst implementing trauma-informed practice involves changes to school systems as a whole, teachers’ attitudes toward trauma-informed practice and their psychosocial competencies, are thought to be important drivers of the day-to-day and moment-to-moment interactions that determine the extent to which the school operates in a trauma-informed way (Baker et al., 2016; Metz et al., 2007). For instance, teachers with positive attitudes toward trauma-informed care are more likely to view each student as being affected in some way by their experience (rather than viewing their behavior as inherently oppositional or defiant) and to respond to students with compassionate and empathy (Thomas et al., 2019).

In addition to shifting attitudes and enhancing knowledge, trauma-informed approaches also emphasize self-care for educators. Self-care in this context involves paying specific attention to the health and wellbeing of teachers and other school staff and acknowledging that supporting adversity-affected students can be a source of amplified teacher stress (Kim et al., 2021).

### Teacher Stress and Burnout

Figley (1995) and Maslach (2003a) observed that there is a cost to caring for helping professionals, and this cost is increasingly evident amongst education staff as many countries report high levels of teacher stress (Education Support, 2019; Foley & Murphy, 2015; Montgomery & Rupp, 2005). Frequently, the risks that teachers face to their professional quality of life are described as burnout, compassion fatigue, and vicarious or secondary traumatization. Stamm (2010, 2012) observed that professional quality of life incorporates significant risks (compassion fatigue) as well as considerable benefits (compassion satisfaction).

Compassion fatigue is the product of bearing witness to the suffering of others resulting in a reduced ability or capacity to be present with others, and feelings of powerlessness, isolation, and confusion (Figley, 2002). Stamm ( 2010) conceptualizes compassion fatigue as consisting of two elements: burnout and secondary traumatic stress. Burnout entails physical and emotional exhaustion, cynicism, and decreased sense of efficacy. It is typically caused by the chronic strain that results from insufficient resources and excessive demands or incongruence between individuals and the work they do (Maslach, 2003b). Secondary traumatic stress occurs when teachers who have direct contact with children’s traumatic stories, exhibit responses similar to those who have experienced trauma first-hand. These responses might include, re-experiencing the trauma through dreams, recollections, and/or flashbacks, avoiding reminders of the trauma through detachment from others, and heightened/persistent arousal evident by difficulty sleeping, becoming irritable or being hyper-vigilant (Figley, 2002). Secondary traumatic stress and burnout are more common when teachers have their own personal experience of trauma, when the demands of work exceed available resources, and when there are feelings of having little control over the quality of services provided (Caringi et al., 2015). Experiencing stress and burnout is obviously distressing for teachers themselves, and it is predictive of negative outcomes for students (Oberle & Schonert-Reichl, 2016).

Figley & Stamm (1996) noted that the key to preventing compassion fatigue lies in professionals detecting and reinforcing the sense of satisfaction they derive from working with clients/students. Stamm (2010) uses the term *compassion satisfaction* to refer to the positive and protective emotional state that one achieves through helping others and feeling success in one’s job. According to Stamm it is “the pleasure you derive from being able to do your work well” (2010, p. 2). Thus, to remain effective and vital in their work, teachers must be able to recognize and find joy in their caring role. Whilst the construct of compassion has been frequently overlooked, there is now growing interest in exploring the role of compassion in professional practice.

### Compassion

Compassion has been defined as “being sensitive to the suffering of self and others, with a deep commitment to try to prevent and relieve it” (Gilbert & Choden, 2015, p. XXV). It has been identified as a promising construct for frontline professionals in terms of its ability to promote psychological wellbeing, as well as increase sensitivity to detect, tolerate and respond to distress in others (Gilbert et al., 2011). Self-compassion entails turning toward our own experience - even when it is painful - and extending understanding, warmth and kindness to oneself (Neff & Germer, 2018). Extending compassion towards ourselves in this way inclines us toward being more giving, caring and supportive in our relationships with others (Gilbert & Choden, 2015).

Neff (2003) conceptualized self-compassion along three dimensions: self-kindness (versus self-judgment), which is the intentional act of extending warmth and understanding when we suffer, fail, or feel inadequate; common humanity (versus isolation), which is about honoring the unavoidable fact that life involves suffering for everyone, without exception; and mindfulness (versus over-identified), defined as being aware of moment-to-moment experience, without judgement. Self-compassion is positively associated with life satisfaction, happiness, optimism, positive affect, wisdom, personal initiative, curiosity, and exploration and negatively associated with depression, anxiety, negative affect, rumination, and thought suppression (Neff, 2003; Neff et al., 2009).

Research with teachers has shown that self-compassion reduces psychological distress and burnout and enhances teachers’ ability to create and maintain optimal classroom environments (Dave et al., 2020; Flook et al., 2013; Jennings, 2014). However, despite the importance of compassion in engaging with the distress, at present, little is known about the role that self-compassion plays in teachers’ attitudes toward trauma-informed practice, nor their overall sense of professional wellbeing.

### Trauma-Informed Practice in Ireland

In recent years, educational policy in Ireland has placed a strong emphasis on whole-school wellbeing promotion and on continuum of support models, which makes provision for both universal and targeted approaches in recognition that children have different needs at different times (Department of Education and Skills, 2019; National Educational Psychological Service, 2007). However, whilst there is growing consensus internationally of the need for trauma-informed approaches in education and other human services sectors, no policy exists in Ireland that explicitly addresses childhood adversity or offers guidance for organizations on responding to the needs of traumatized children (Prevention and Early Intervention Network, 2019).

Despite the absence of explicit policies and resources in the area, many education professionals have recognized the need within their own schools and have begun researching and implementing trauma-informed approaches of their own volition. Several continuing professional development courses have been made available in response to demand from educators, and a pilot Nurture Schools project was launched in 2020 across a network of schools (Educate Together Nurture Schools Project). Thus, the impetus for trauma-informed approaches in Ireland is emerging in an organic, bottom-up fashion, as practitioners seek ways to respond to the strengths and needs within their own school communities. However, because these bottom-up changes have not, thus far, been met by a top-down commitment in legislation, policy or resourcing, efforts to embed trauma-informed approaches are fragmented and largely based on the commitment and resourcefulness of individual school leaders. Overall awareness of trauma-informed approaches across the education sector remains limited. Furthermore, whilst there have been some studies of teacher stress in Ireland (Darmondy & Smyth, 2011; Foley & Murphy 2015; INTO, 2015), there is a gap in research on teacher quality of life and compassion within the Irish context.

### The Current Study

Against this education policy and practice backdrop, the primary purpose of this study was to assess teachers’ attitudes, dispositions, and readiness for implementing trauma-informed approaches. The study also examined teachers’ professional quality of life and self-compassion. It sought to investigate whether compassion mediates the experience of burnout and secondary-traumatic stress, and contributes to a willingness to engage with, and respond to, student distress as would be required by trauma-informed approaches. Teachers completed the Professional Quality of Life Scale (ProQOL), which measures secondary traumatic stress, burnout, and compassion satisfaction, as well as the Self-Compassion Scale and the Attitudes and Readiness for Trauma-Informed Care (ARTIC) Scale. They also provided personal details (gender, age, experience) and school characteristics (sector, geographical location, socio-demographic profile). We hypothesized that teachers would demonstrate broadly positive attitudes toward trauma-informed practice and that higher self-compassion and compassion satisfaction would be associated with more positive views of trauma-informed practice.

## Methodology

### Study Design and Procedure

This study explored correlations between teachers’ attitudes to trauma-informed care, their professional quality of life and their levels of self-compassion. This study was conducted in line with the principles of the Declaration of Helsinki and ethical approval was obtained from the lead author’s University Ethics Committee. Data were collected via a secure online survey site. The survey consisted of questions pertaining to participant demographics and school characteristics, along with three standardized scales (described below). All data were collected mid-February to March 2020, just prior to the emergence of the Covid-19 pandemic in Ireland. The first Covid-related lockdown saw schools in Ireland close on March 12th 2020 for the remainder of the academic year; the vast majority (98%) of participants had responded prior to this.

### Participants

Participants were 377 primary and secondary school teachers in the Republic of Ireland. An invitation to participate was sent to professional teaching bodies in Ireland and distributed on social media (Twitter), along with a link that took participants directly to the online survey. The convenience sample was between 22 and 63 years old (*M* = 40.6, *SD* = 9.4). The majority of participants were female (77%, *n* = 289). On average, participants had 15.8 years teaching experience (*SD* = 9.2). As detailed in Table 1, the majority of participants taught in a secondary school in an urban area or a town. Most schools were publicly funded, had a mixed gender intake, and were not located in areas of social disadvantage.

**Table 1:** Sample Demographics

Category	Variable	*n*	%
Gender	male	88	23.3
	female	289	76.7
Sector	Primary School	89	23.6
	Secondary School	272	72.1
	Other^a^	16	4.2
DEIS^b^ Status	DEIS	96	25.5
	Non-DEIS	281	74.5
Location	Urban	151	40.1
	Rural	76	20.2
	Suburban/Town	150	39.8
Funding	Private	17	4.5
	Public	360	95.5
Gender Mix	Single Sex Girls	52	13.8
	Single Sex Boys	41	10.9
	Mixed	284	75.3
Role	Classroom Teacher	204	54.3
	Principal/Deputy Principal	43	11.4
	Post of Responsibility (incl. Assistant Principal or Year Head)	50	13.3
	Special Education, Learning Support or Resource Teacher	53	14.1
	Guidance Counsellor	13	3.4
	Other^a^	14	3.5

^a^ The ‘Other’ category refers to participants working in other areas of education, including Higher and Further Education, Youth Reach, Preschool, Home-School Liaison, and one case no role/sector information was provided

^b^ DEIS (Delivering Equality of Opportunity in Schools) refers to schools that qualify for entry into a government funded scheme that provides additional resources for schools with high concentrations of students from socioeconomically disadvantaged backgrounds (DES, 2005)

### Measures

In addition to demographics information, the survey included three validated measures: the Professional Quality of Life Scale - version 5 (Stamm, 2010), the Self-Compassion Scale (Neff, 2003), and the Attitudes Related to Trauma-Informed Care Scale (Baker et al., 2016). The Professional Quality of Life Scale (ProQOL5) is a 30-item scale, which prompts participants to reflect on relationships and compassion towards people they work with. It comprises two constructs: Compassion Satisfaction (CS) and Compassion Fatigue (CF). CS describes the positive aspects of one’s professional role; the degree of pleasure derived from doing your job well. CF describes the negative aspects – it is comprised of Burnout (BO) and Secondary Traumatic Stress (STS). BO is associated with a loss of hope and difficulties carrying out work duties effectively. STS measures exposure to the trauma of others through work. The scale has been used in a variety of contexts internationally and has been psychometrically validated (Stamm, 2010). Cronbach’s alpha levels observed for the current study were CS: 0.91, BO: 0.83, and STS: 0.82.

The Self-Compassion Scale (SCS) is a 26-item questionnaire that provides an overall self-compassion score and six subscale scores. Self-compassion entails three key components, each of which has a positive and negative pole, thereby forming six subscales: self-kindness versus self-judgment, a sense of common humanity versus isolation, and mindfulness versus over-identification. The scale uses a 5-point Likert scale and participants are asked to indicate for each item how often they behave in the manner described. Cronbach’s alpha levels observed for the current study were: SCS total 0.91, self-kindness 0.80, self-judgement 0.79, common humanity 0.76, isolation 0.77, mindfulness 0.71 and overidentification 0.72.

The Attitudes Related to Trauma-Informed Care (ARTIC-35) was used to measure participants’ attitudes towards trauma-informed care in relation to their work. This 35-item scale can be used in educational settings that have not yet implemented trauma-informed approaches. The language is adjusted to suit the context, such that the term *student* is used rather than *client*. The measure uses a 7-point scale in between two contrasting statements, and participants are asked to indicate where they would place themselves on this continuum. It consists of five subscales, these are: (1) underlying causes of problem behavior and symptoms, which probes beliefs around behavior and symptoms as fixed entities or adaptation to current contexts; (2) responses to problem behavior and symptoms, which probes a commitment to regarding positive relationships as a means of change; (3) on-the-job behavior, which probes empathy versus control-focused staff behavior; (4) self-efficacy at work, which probes participants’ perception of ability to meet demands of students; and (5) reactions to work, which probes the engagement with secondary trauma and support-seeking behaviors. Internal consistency of the measure was high with Cronbach’s alpha = 0.88 overall.

## Results

**Fig. 1:**
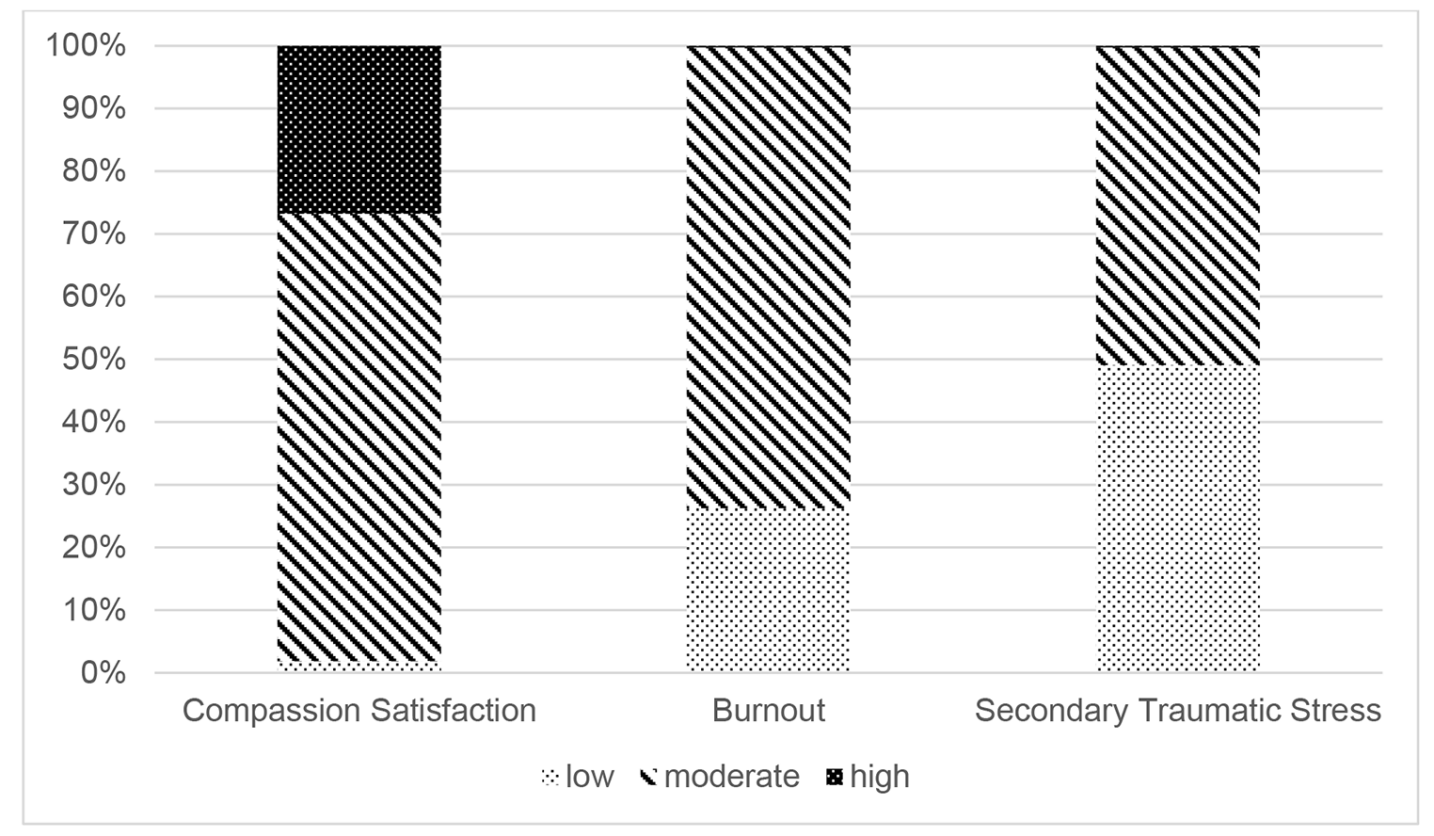
Participant scores on the Professional Quality of Life subscales

Mean scores for each of the three measures employed in the study are displayed in Table 2. On the ProQOL5, participants scored highly on the Compassion Satisfaction subscale, which indicates they tend to find meaning and purpose in their work. Fewer than 2% (*n* = 7) of participants scored within the low range on this measure (see Fig. 1). On the Burnout subscale, nearly three-quarters of participants (73.5%, *n* = 277) reported moderate levels, around a quarter (26.3%, *n* = 99) reported low levels, and just one participant (0.3%) reported a high level of burnout. About half of participants (49.1%, *n* = 185) reported low levels of Secondary Traumatic Stress, with the other half (50.7%, *n* = 191) reporting moderate levels, and one participant (0.3%, *n* = 1) reporting a high level.

**Table 2:** Mean and Standard Definition of Measures

	Mean(SD)
Professional Quality of Life	
Compassion Satisfaction	37.55(6.40)
Burnout	26.80(6.09)
Secondary Traumatic Stress	23.44(6.08)
Self-Compassion Scale	
Self-Kindness	2.97(0.77)
Self-Judgement	3.18(0.84)
Common Humanity	3.33(0.81)
Isolation	3.15(0.71)
Mindfulness	3.27(0.73)
Overidentified	3.04(0.85)
*SCS Total*	3.05(0.61)
ARTIC	
Underlying Causes of Problem Behavior and Symptoms	4.63(0.84)
Responses to Problem Behavior and Symptoms	5.21(0.88)
On-The-Job Behavior	5.28(0.78)
Self-Efficacy at Work	4.90(0.96)
Reactions to the Work	5.04(0.84)
*ARTIC Total*	5.01(0.65)

**Fig. 2:**
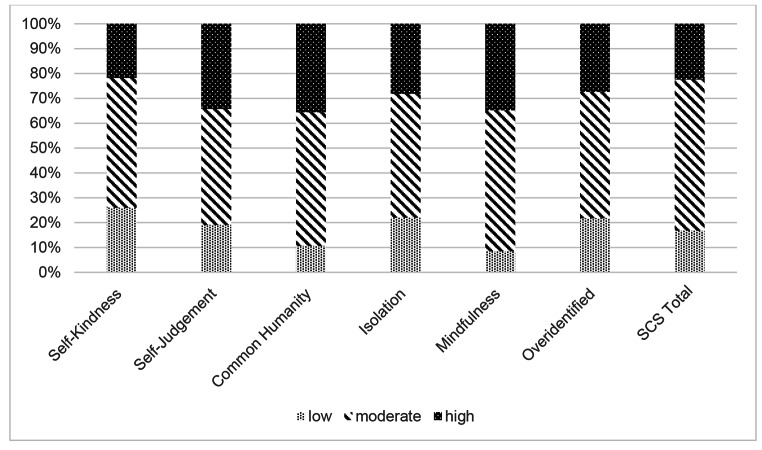
Breakdown of scores within the low, moderate or high range on the Self-Compassion scale and subscales

On the Self-Compassion Scale (SCS), nearly two-thirds of participants (60.8%, *n* = 228) scored between 2.5 and 3.5, which is considered the moderate range. Sixty-three participants (16.7%) were low in self-compassion, and 22.3% (*n* = 84) reported high self-compassion scores. A breakdown of scores across the six subscales is presented in Fig. 2. The average score on the ARTIC-35 was 5.01 (*SD* = 0.65). This constitutes a score above the mid-point of 4, indicating favorable attitudes towards trauma-informed care.

As displayed in Table 3 all three measures were correlated. There was a strong positive correlation between the ARTIC and Compassion Satisfaction (0.53). High ARTIC-35 scores are associated with lower Burnout (-0.41) and lower Secondary Traumatic Stress scores (-0.20). There was also a positive correlation (0.31) between the ARTIC and the SCS.

**Table 3:** Correlations between Professional Quality of Life, Self-Compassion, and Attitudes to TIC

	1	2	3	4	5
1 Compassion Satisfaction	1				
2 Burnout	− 0.672^*^	1			
3 Secondary Traumatic Stress	− 0.341^*^	0.668^*^	1		
4 Self-Compassion Scale	0.305^*^	− 0.450^*^	− 0.376^*^	1	
5 ARTIC-35	0.531^*^	− 0.413^*^	− 0.195^*^	0.313^*^	1

Note. Items 1–3 are ProQOL subscales

*Correlations are significant with *p* < .01

A hierarchical regression analysis was performed to explore the relationship between demographic variables, professional quality of life, self-compassion, and attitudes to trauma-informed care. Variables are entered stepwise to allow for a comparison of the unique contribution and mediating effects of different blocks of variables. In model 1, gender and age were entered into the model to ascertain the extent to which these variables can predict ARTIC-35 scores. In model 2, school characteristics were added. Model 3 includes self-compassion scores, and finally, model 4 includes Professional Quality of Life scores. This final model with all variables included explains 34.2% of the variance in ARTIC-35 scores. About half of this is explained by the addition of the three ProQOL subscales (Model 4 in Table 4). In the final model (Table 5), age remains a significant predictor, with older teachers showing more positive attitudes, and teaching in a single-sex boys’ school predicted less favorable attitudes. Further, self-compassion and compassion satisfaction were strong predictors of more positive attitudes toward trauma-informed care.

**Table 4:** Hierarchical regression model summary of ARTIC-35 score predictors

Model	Adjusted *R*^*2*^	*SE* of the Estimate	Sig. *F* change
1 Gender + Age	0.027	0.65	*p =* .002
2 + School Characteristics	0.083	0.63	*p =* .001
3 + SCS	0.168	0.60	*p <* .001
4 + ProQOL	0.342	0.53	*p <* .001

*Note. N* = 370 included in the regression. School characteristics include role, sector, school type and area. Full list of included variables displayed in Table 5

**Table 5:** Predictors of ARTIC-35 scores

	*B*	SE *B*	β	t	p
Intercept	2.86	0.46		6.26	< 0.001
**Age**	0.01	0.003	0.12	2.38	0.010
gender = male (ref: female)	-0.07	0.07	-0.04	-0.97	0.331
Role (ref: Classroom Teacher)					
Principal/Deputy Principal	0.13	0.10	0.06	1.33	0.184
Post of Responsibility	0.05	0.09	0.02	0.52	0.602
Special Ed/Support/Resource	0.17	0.09	0.09	1.94	0.053
Guidance Counsellor	0.16	0.16	0.05	1.02	0.307
Other	0.38	0.27	0.11	1.40	0.162
Sector (ref: Secondary School)					
Primary School	0.12	0.07	0.08	1.60	0.111
Other	-0.28	0.25	-0.09	-1.15	0.249
Gender mix (ref: co-ed)					
Single-sex girls	-0.04	0.09	-0.02	-0.45	0.655
**Single-sex boys**	-0.20	0.01	-0.10	-2.08	0.039
Private school (ref: public)	0.17	0.15	0.05	1.15	0.251
DEIS (ref: non-DEIS)	0.11	0.07	0.07	1.54	0.125
Area (ref: urban)					
Rural	-0.15	0.08	-0.09	-1.80	0.073
Suburban/Town	-0.07	0.07	-0.05	-0.96	0.337
**Self-Compassion**	0.15	0.05	0.13	2.72	0.007
**Compassion Satisfaction**	0.04	0.01	0.40	6.37	< 0.001
Burnout	-0.01	0.01	-0.11	-1.39	0.166
Secondary Traumatic Stress	0.01	0.01	0.06	0.95	0.344

*Note.* N = 370. Statistically significant indicators are in bold

## Discussion

The results show that in general, teachers display an orientation towards their students and towards themselves that is conducive to trauma-informed practice. Positive dispositions were evident across all five subscales of the ARTIC and the total score. Overall, teachers had moderate levels of self-compassion. They exhibited low to moderate levels of secondary traumatic stress and burnout, and notably high levels of compassion satisfaction, indicating that they tend to embrace their caring role and find meaning and purpose in their work. Furthermore, compassion satisfaction was the strongest predictor of positive attitudes toward trauma-informed care, followed by self-compassion.

The results in relation to compassion satisfaction are generally positive and in line with previous findings demonstrating that an ethic of care and a desire to sustain positive interpersonal relationships are central drivers in teachers’ practices (O’Toole & Simovska, 2021). However, the ARTIC results recorded in this study were lower overall compared to those involving other research participants and in other jurisdictions, including frontline health and social care professionals in Ireland (O’Toole & Dobutowitsch, 2020), educators in the United States (Kim et al., 2021), residential care professionals in Australia (Galvin et al., 2020), and student teachers in Canada (Rodger et al., 2020). All of these studies reported average or median ARTIC scores between 5.08 and 6.10. These findings are perhaps unsurprising considering the lack of acknowledgement of childhood adversity and trauma-informed practice within Irish educational policy landscape. It should be noted however, that meaningful comparison across studies is difficult, since norms/benchmarks for the ARTIC scale are not currently available. In addition, some of the studies mentioned above used the ARTIC to assess attitudes following the provision of professional development training. At the time of data collection for this study, teachers had extremely limited access to trauma-informed professional development programs. The results suggest a need for greater awareness of trauma-informed practice and enhanced capacity building in the Irish context. They also suggest that these measures would be positively received, given teachers’ self-reported capacities and dispositions.

The relationship between quality of life, self-compassion, and attitudes towards trauma-informed care were weak to moderate overall but showed a pattern whereby positive attitudes toward trauma-informed care were associated with higher compassion satisfaction and self-compassion, less burnout and less secondary traumatic stress. The compassion constructs (compassion satisfaction and self-compassion) were the most significant predictors of positive attitudes toward trauma-informed practice and remained significant even when other factors were controlled for. These findings correspond with previous research. For instance, Jennings (2015) found that higher levels of teacher self-compassion were associated with greater classroom emotional support. Christian-Brandt et al., (2020) found that high levels of compassion satisfaction were associated with perceived effectiveness of trauma-informed care. Whilst further research is needed to determine causality, these results suggest that compassion plays an important role in teachers’ professional wellbeing and creating optimal environments to support students impacted by trauma.

The results also showed some interesting variations on the self-compassion subscales. As noted in the Introduction, self-compassion is comprised of three elements (kindness, common humanity, and mindfulness), each of which has a positive and negative pole (Neff, 2003). This study found that teachers display a healthy tendency toward mindfulness and common humanity, yet extending kindness towards themselves seems harder. Thus, teachers may benefit from having opportunities to engage in experiential compassion-based practices, which could be done informally through teacher-led communities of practice, and/or through more formal and structured programs.

Regarding demographic and school variables, this study found that gender, the educational role of participants, and school sector (primary or secondary) did not predict attitudes toward trauma-informed care; nor did the disadvantage (DEIS) status or geographical location of the school. However, older teachers were more likely to display positive attitudes toward trauma-informed care, whilst teachers in single-sex boys’ schools held attitudes that were less favorable. Our finding in relation to teachers’ age conflicts with that of Christian-Brandt and colleagues (2020), who found that older teachers displayed *less positive* views regarding the perceived effectiveness of trauma-informed care. However, our finding corresponds with existing research within the Irish context. The ARTIC assesses the various responses and dispositions of educators to students experiencing emotional distress and behavioral difficulties. Previous studies in Ireland have highlighted that responding to students with emotional and behavioral difficulties is one of the biggest concerns of teachers in the early stages of their careers, who are likely to be younger (Clarke et al., 2012; O’Toole & Burke, 2013). This suggests there is a need for greater support for trainee and newly qualified teachers. Whilst the past decade has seen substantial reform in teacher education at all stages of the continuum - from initial teacher education, induction and continuing professional development (Harford & O’Doherty, 2016) - questions remain about how teachers are prepared for the complexity of children’s emotional and behavioral needs (Darmody & Smyth, 2011). To date, behavior management approaches emphasizing external control of children’s behavior through positive and negative consequences have dominated in schools, despite growing calls for more relationship-oriented and restorative approaches, which are more likely to engender supportive learning communities and promote student autonomy (Brophy, 2006; Brummer, 2020).

Results showed that teachers in single-sex boys’ schools held less positive attitudes toward trauma-informed practice. Previous authors have argued that these schools can reinforce hypermasculine and heteronormative discourses, potentially cultivating a culture of ‘toxic masculinity’ (Cushman, 2010; Hickey & Mooney, 2018). The attitudes and dispositions required for trauma-informed practice (care, compassion, emotional understanding) are entirely at odds with myths surrounding dominant masculinity (e.g., toughness, dominance, emotional insensitivity). It has been noted that school personnel can be ‘sucked into’ the prevailing culture of their school. For instance, Burke (2011) describes how staff became tolerant of - and even complicit in - reinforcing dominant masculine norms in one all-boys’ school. Thus, it seems possible that our participants were influenced by the culture that pervades in all-boys’ schools and that this negatively impacted their attitudes toward trauma-informed practice. Additional research is needed to investigate this further. However, it is noteworthy that compared to European counterparts, Ireland has a uniquely high number of gender-segregated school (one-third of all second-level students and 17% of primary-level pupils attend a single-sex schools). The current results reinforce concerns about gender-segregated education in the Irish context.

This study found moderate levels of burnout and secondary traumatic stress were typical for Irish educators. Data gathering for this study occurred just prior to the emergence of the Covid-19 pandemic in Ireland. Research has shown that the pandemic has increased the levels of stress for both educators and children. For instance, during lockdowns, teachers were required to rapidly adapt their ways of working often without sufficient resources or training. Many teachers in Ireland and elsewhere felt a deep sense of frustration and anger at perceived lack of support from central educational authorities (Kim & Asbury, 2020; O’Toole & Simovska, 2021). The pandemic has also exposed children to unprecedented disruption, and whilst some children may have benefited from increased interactions with parents and siblings during periods of lockdown, many others have experience elevated emotional distress and are at heightened risk for experiencing domestic violence, emotional, physical, and sexual abuse, and economic hardship (Hamoda et al., 2021; Mahase, 2020; Marmot et al., 2020; United Nations, 2020).

With evidence of increased distress and trauma experienced by children during the pandemic, it is clear that a commitment to resourcing trauma-informed approaches in schools is needed now, more than ever. Furthermore, teachers were dealing with the professional and personal impact of the pandemic on their own lives, but also acutely aware of the inequalities and adversities faced by their students, with many remaining devoted to their students during periods of lockdown, sometimes with the cost of increased stress, vulnerability or concern for themselves or their own families (O’Toole & Simovska, 2021). Previous authors have argued that support for teachers is the “critical missing ingredient” from school trauma-informed efforts (Luthar & Mendes, 2020, p. 153). In future trauma-informed work, it will be essential that teacher wellbeing is prioritized and supported systemically, as it is clear that teacher and student wellbeing are co-dependent and intimately entangled (O’Toole, 2022b).

There is a need, therefore, for greater systemic support for nurturing compassion and trauma-informed approaches in education. This might include making provision for compassion-focused practices (as noted above), but it will also be important that compassion and relationship-oriented practices are embedded and embodied within education systems in a holistic way (Treisman, 2017). Senge ( 1990) argues that systems thinking is the conceptual cornerstone of any learning organization. In relation to schools, a systems approach necessitates a whole-school perspective; seeing the dynamic and relational processes, rather than focusing solely on individuals or discrete parts. It means facilitating dialogue to build a shared vision, and taking collective informed and committed actions for change. Senge and colleagues (2019) have developed the Compassionate Systems Framework in Schools, the aim of which is to grow “compassionate integrity” in students and teachers - fostering awareness of human inter-connectedness; which they argue is vital to human prosperity and even survival, especially in a world grappling with immense environmental, political and socioeconomic challenges.

Another concrete recommendation is for the provision of professional reflective supervision to be made available to teachers. Professional supervision is an integral component of many professions that involve the care and protection of children and young people, including social work, counselling and clinical practice. It involves dedicated time for reflection and confidential emotional support with a trusted colleague. Lawrence (2020) found that professional supervision was highly desired amongst education staff in the United Kingdom and whilst acknowledging practical challenges and resource implications, these were not deemed insurmountable.

In sum, whilst there can be much additional stress from routinely providing empathic support to trauma-affected students, teachers in this study demonstrated the courage and commitment to care. They exhibited generally positive dispositions towards, and a readiness for implementing trauma-informed practice, despite a lack of resourcing and policy commitment in the area. This study highlights the need for policymakers in Ireland to ensure dedicated resources to support the uptake of trauma-informed practice in schools. It also underscores the centrality of compassion in trauma-informed practice, and points to the importance of nurturing compassion in the education system, to ensure all members of the school community feel seen, heard, valued, and cared for.

## Limitations and Future Directions

The study is cross-sectional, relies on self-report, and used a convenience sample; no causal inference can be made about associations between compassion and trauma-informed practice. It is possible that there is self-selection bias, and that the data reflect the views and attitudes of individuals with an interest in the topic and prior training. However, there are no indicators to suggest that the data are skewed due to a systematic bias. It is also worth noting that the ARTIC is only an indicator of attitudes; it does not assess whether teachers’ behaviors are consistent with their attitudes. Furthermore, the views of individual teachers may not be in concordance with school-wide ethos or values in which they work. Future studies should consider organizational processes of the school as a whole, including the barriers and facilitators to the co-creation, implementation and sustainment of compassion and trauma-informed approaches.
