# Correction To: The Courage to Care: Teacher Compassion Predicts more Positive Attitudes Toward Trauma-Informed Practice

**DOI:** 10.1007/s40653-022-00492-z

**Published:** 2022-11-02

**Authors:** Catriona O’Toole, Mira Dobutowisch

**Affiliations:** 1 Department of Education, Maynooth University, Maynooth, Co Kildare Ireland; 2 Marino Institute of Education, Griffith Avenue, Dublin 9, Dublin, Ireland

**Correction to: Journal of Child & Adolescent Trauma**

 10.1007/s40653-022-00486-x

The original online version of this article was revised. The surname of the second author was misspelled. Correct info below. Also, Figures 1 and 2 are difficult to read in black and white, textured graphs can be found below.

Catriona O’Toole

Mira Dobutowitsch

**Fig. 1:**
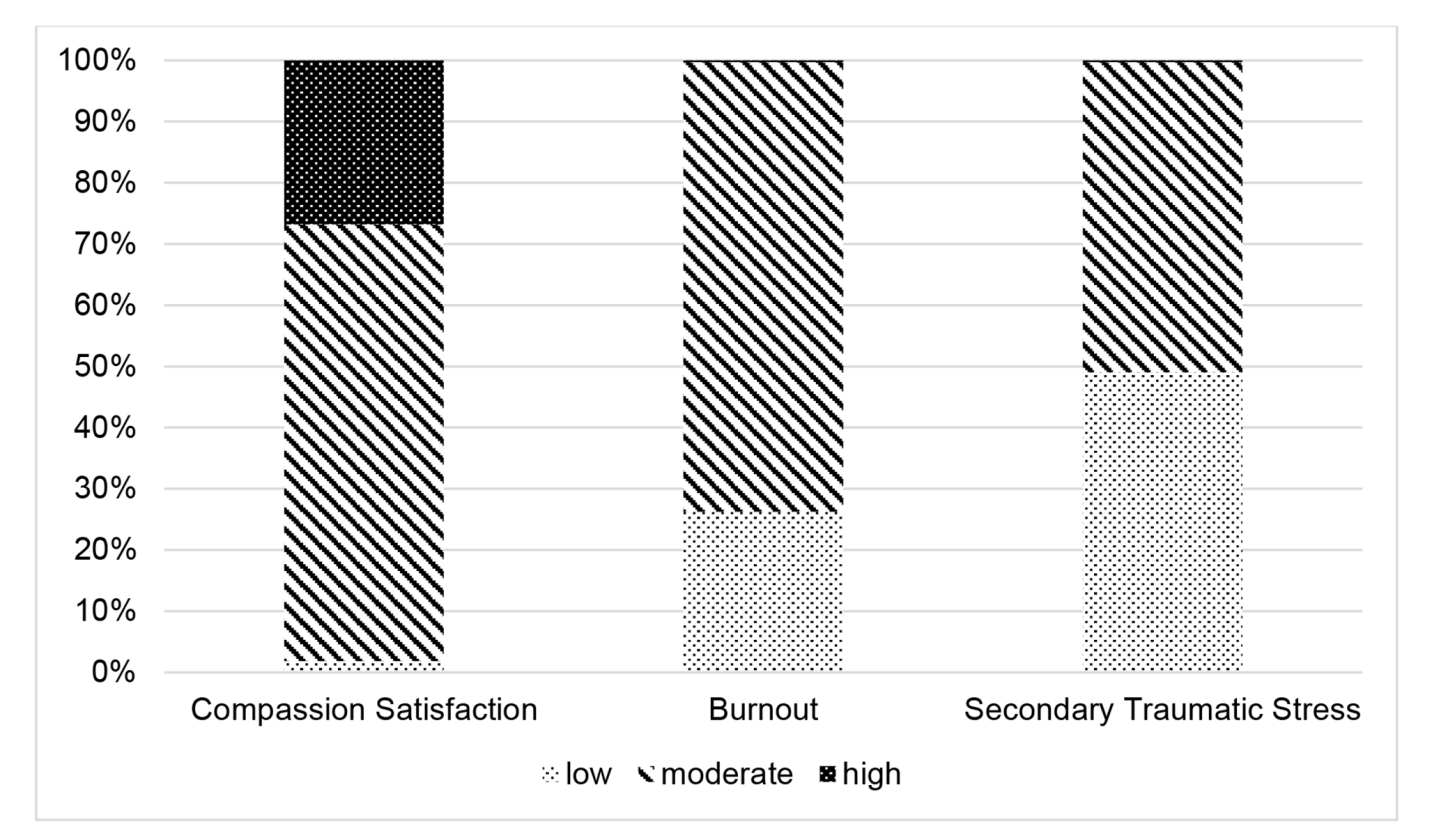
Participant scores on the professional quality of life subscales

**Fig. 2:**
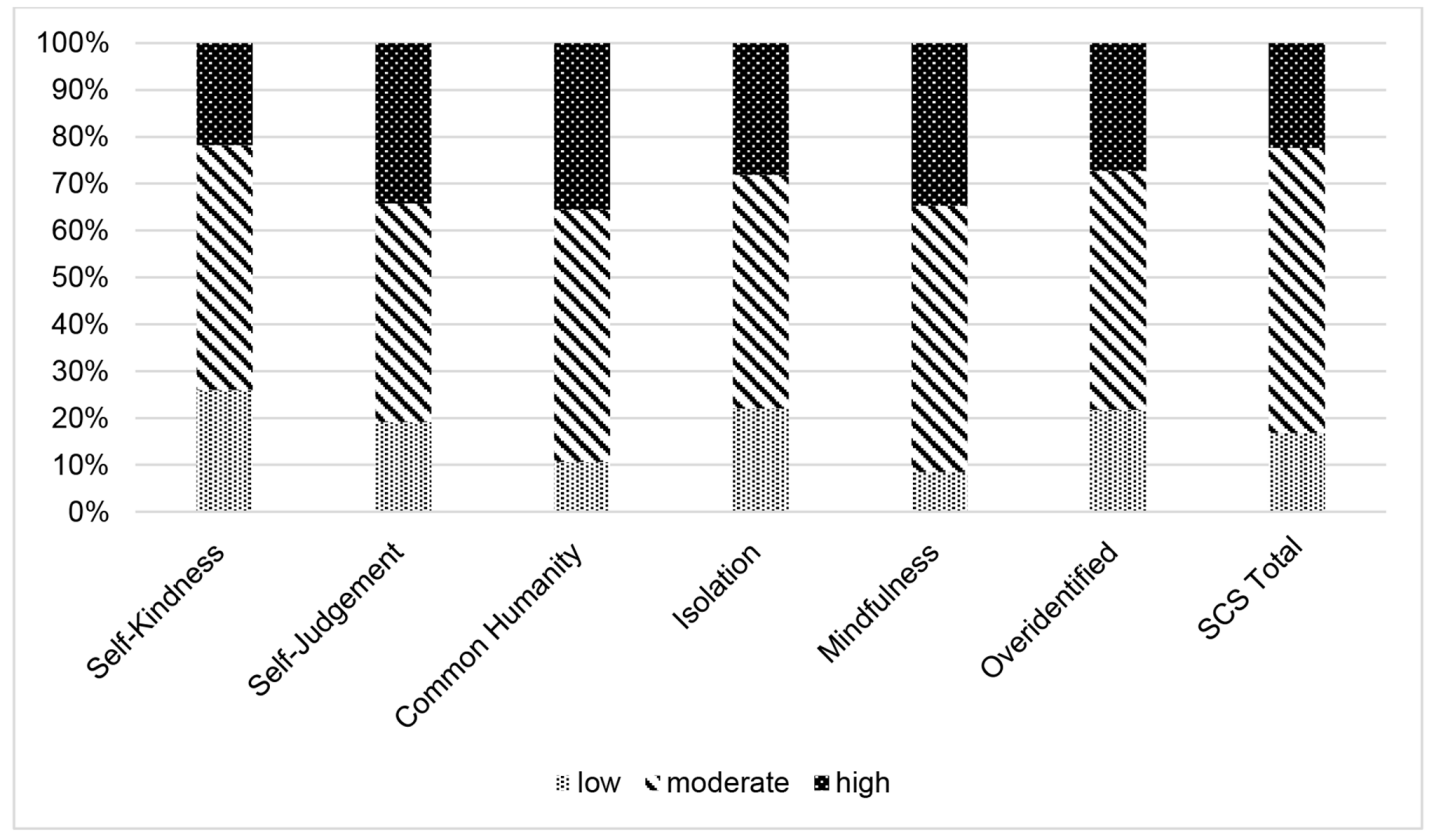
Breakdown of scores within the low, moderate or high range on the self-Compassion scale and subscales

